# Reports of Contagious Diseases to the Board of Health

**Published:** 1885-02

**Authors:** 


### Editorial.

REPORTS OF CONTAGIOUS DISEASES TO THE BOARD OF HEALTH.

Our City Board of Health is unusually active at the present time, and never before, in the history of Buffalo, has the vigorous action of this body been more necessary. While the city, to the superficial observer, may appear to be in fair sanitary condition, in reality there are but few towns in the Union that contain a larger proportion of disease-breeding hot-beds. There are tenements swarming with inhabitants, many of whom sleep in rooms that the light of heaven has never seen; water-closets reeking with filth and exhaling deadly miasms; yards filled with putrefying heaps of garbage that have been accumulating for months, and whole districts that are not properly sewered or supplied with wholesome water.

While the sanitary authorities are doing all in their power to abate nuisances of this character, they will, at the same time, adopt new measures to arrest the progress of contagious and infectious diseases.

Blank cards have been sent out to the physicians requiring a report of the contagious diseases coming under their observation. These reports are to be given to the heads of families, who will deliver the same to the clerk of the Board of Health within twenty-four hours. If deemed necessary, the Board of Health will cause a placard denoting the character of the disease to be placed upon the house in which the infectious disease exists.

This plan has been successfully carried out in Milwaukee and Detroit for the past six years and has recently been adopted in Rochester and other cities of this State.

The objections to the plan are obvious but not insuperable.

1. Many physicians, on account of carelessness or ignorance, will object to this addition to their already numerous duties. But the work required is slight, and no intelligent practitioner will fail to see the importance of the matter. If any one should be so perverse as to refuse to do his duty in this respect, a prosecution by the City Attorney would serve to enlighten him.

2. People will object to having their houses placarded as an infringement upon their personal liberty. But people do not object to have small-pox signs placed upon their houses. Why should they object to publishing the presence of diphtheria and scarlet fever, which number their victims by thousands to the hundreds destroyed by small-pox?

But the advantages of this plan are so great as to more than counterbalance these objections.

1. The householder is protected against prosecution by law for allowing a person to become infected on his premises without due warning of the danger. It has been decided by the courts that "If a man has contagious disease in his house, and fails to give due notice thereof, or allows people to come into his house without sufficient warning, or in any avoidable way exposes the public to contagion on his own premises, he is liable to indictment at the common law." (People vs. Townsend, 3 Hill, N. Y., 479; Welch vs. Stowell, 2 Doug. Mich., 332, etc.) "It is held that where a person knowingly communicates a contagious disease to another, and death results therefrom, the crime is manslaughter." (*Medico-Legal Journal*, Vol. I., No. 3, p. 394.)

2. By reporting contagious diseases to the Board of Health, their extent and location can be definitely known, and the percentage of mortality can be definitely ascertained, thus affording a valuable means of studying the relations of various sanitary conditions to the progress and mortality of each disease.

3. The spread of these diseases will be greatly diminished. Children coming from infected houses can be rejected from school, and all who can read will avoid the dangerous premises.

4. The health authorities are enabled to provide for the prompt removal of the dead and the thorough disinfection of the infected premises. During the past summer we saw the body of a child, dead with malignant scarlet fever, kept for two days in rooms inhabited by other children, and within ten days every child in the house died of the same disease. Such a crime as this should no more be tolerated than infanticide. By compelling the prompt removal of the dead and the suppression of public funerals, many of these horrible consequences could be avoided.

